# Prescription for Dysmenorrhœa

**Published:** 1879-12

**Authors:** 


Prescription for Dysmenorrhœa.—In a note in the *Trnsactions of the Ohio Medical Society*, 1879, we find that Dr. W. H. Mussey, of Cincinnati, during a discussion on dysmenorrhœa, stated the following formula, which he has found "very efficacious in the treatment of all forms of dysmenorrœa:"

℞—Pulveris guaiaci resinæ
Terebinthinæ canadensis................aa℥j
Olei sassafras..........................ʒij
Alcoholis..............................℥viij
M. Macerate for seven days and then strain. Then add:
Hydrargyri chloridi corrosivi...........℈j

Mix well, and Sig: Take twenty drops, in wine or sweetened water, every night and morning.

